# Development of a cellulose-based prosthetic mesh for pelvic organ prolapse treatment: *In vivo* long-term evaluation in an ewe vagina model

**DOI:** 10.1016/j.mtbio.2021.100172

**Published:** 2021-11-27

**Authors:** Chen Lai, Shu-Jiang Zhang, Xuan-Chen Chen, Li-Yuan Sheng, Tian-Wei Qi, Le-Ping Yan

**Affiliations:** a Shenzhen Key Laboratory of Human Tissue Regeneration and Repair, Shenzhen Institute Peking University, Shenzhen, 518057, PR China; b The First Affiliated Hospital of Guangzhou Medical University, Guangzhou, 510120, PR China; c Faculty of Engineering Science, Technical University of Dresden, Dresden, 01069, Germany; d The Third Affiliated Hospital of Shenzhen University, Shenzhen, 518001, PR China; e Guangdong Provincial Key Laboratory of Digestive Cancer Research, The Seventh Affiliated Hospital of Sun Yat-sen University, Shenzhen, 518107, PR China; f Scientific Research Center, The Seventh Affiliated Hospital of Sun Yat-sen University, Shenzhen, 518107, PR China

**Keywords:** BC, bacterial cellulose, PP, polypropylene, PTFE, polytetrafluoroethylene, POP, pelvic organ prolapse, BCCOL, collagen-coated bacterial cellulose, Pelvic organ prolapse, Cellulose-based vaginal mesh, In vivo evaluation, Tissue integration, Inflammatory reaction

## Abstract

The use of vaginal surgical mesh to treat pelvic organ prolapse (POP) has been associated with high rates of mesh-related complications. In the present study, we prepared new kinds of meshes based on bacterial cellulose (BC) and collagen-coated BC (BCCOL) using a laser cutting method and perforation technique. The mechanical properties of pre-implanted BC meshes, including breaking strength, suture strength and rigidity, were equal to or exceeded those of available clinically used polypropylene meshes. An *in vitro* cellular assay revealed that BCCOL meshes exhibited enhanced biocompatibility by increasing collagen secretion and cell adhesion. Both BC and BCCOL meshes only caused weak inflammation and were surrounded by newly formed connective tissue composed of type I collagen after implantation in a rabbit subcutaneous model for one week, demonstrating that the novel mesh is fully biocompatible and can integrate into surrounding tissues. Furthermore, a long-term (ninety days) ewe vaginal implantation model was used to evaluate foreign body reactions and suitability of BC and BCCOL meshes as vaginal meshes. The results showed that the tissue surrounding the BC meshes returned to its original physiology as muscle tissue, indicating the excellent integration of BC meshes into the surrounding tissues without triggering severe local inflammatory response post-implantation. The collagen coating appeared to induce a chronic inflammatory response due to glutaraldehyde remnants. The present exploratory research demonstrated that the developed BC mesh might be a suitable candidate for treating POP.

## Graphical abstract

**Figure:**
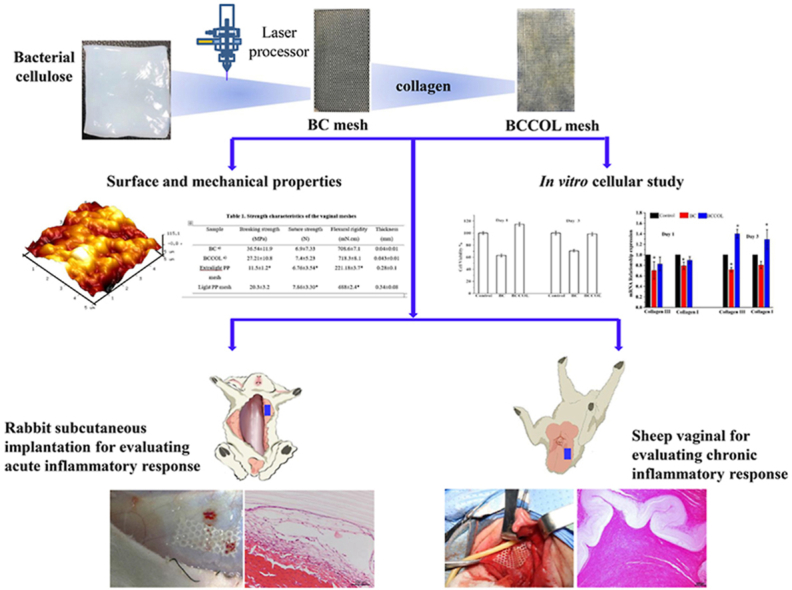

## 1 Introduction

Pelvic organ prolapse (POP) occurs when the tissue and muscles of the pelvic floor no longer support the pelvic organs, resulting in a drop (prolapse) of the pelvic organs (vagina, cervix, etc.) from their normal position. Approximately 35% of parous women have certain degree of POP [1]. Reconstructive surgery is the main treatment for POP. The number of women POP surgery is expected to increase from 166,000 in 2010 to 245,970 in 2050 only in the United States [2]. Surgical mesh is a medical device that is used to provide additional support when repairing weakened or damaged tissue. The US Food and Drug Administration (FDA) approved surgical mesh for use in POP surgery in 2002. The majority of surgical mesh devices currently available for use are prepared from synthetic materials or animal tissue. Mesh made from animal tissue is absorbable and degraded by enzymes *in vivo*, leading to mechanical failure and not able for long-term reinforcement to the repair site, thus they are less commonly used clinically. A range of synthetic materials, including polypropylene (PP) [3,4], polyester (PE) [5], polytetrafluoroethylene (PTFE) [6], have also been used in the clinic.

Among the varieties available, PP is the dominant nonabsorbable material used, as it provides permanent strength reinforcement for urogynecological repair [7]. However, the use of transvaginal meshes to augment pelvic floor repair remains controversial, as reports about serious complications associated with the use of meshes are growing annually. These complications include persistent vaginal bleeding or discharge, pelvic or groin pain, or pain during sex. A reoperation ratio of nearly 30% has been reported [8], and the recurrence risk is as high as 33–45% [9]. In 2011, FDA published a safety communication in response to concerns over surgical complications involving the use of transvaginal mesh for POP [10], and FDA ultimately banned the production and sale of these devices on April 16, 2019 [11]. Additionally, several countries (e.g., Australia and New Zealand) and companies have recently banned or paused the use of synthetic meshes [12]. Currently, these meshes are facing unprecedented challenges. Numerous studies have examined the host response to vaginal meshes, including erosion, shrinkage and adhesion [[13], [14], [15]]. However, what triggers these complications still remains unknown.

Scientists are trying to develop novel meshes for POP treatment by using various biomaterials [16,17]. Knitted polyamide mesh was be developed to deliver endometrial mesenchymal stem cells to improve mesh biocompatibility and restore strength to prolapsed vaginal tissue [16]. Using a modified spinneret electrospinning system, tropoelastin: PCL yarns could be fabricated. This mesh showed excellent integration with new collagen deposition with few pro-inflammatory M1 macrophages after 1 month [17]. Bacterial cellulose (BC), a polysaccharide, is a new type of biomaterial that has been receiving increased attention in recent years. BC is a form of cellulose produced by bacteria [18]. Our group has studied BC-based biomaterials for many years and discovered their potential for use as a surgical mesh [19,20]. BC is advantageous in contrast to native cellulose from plants, as it possesses superior tensile strength and stiffness (as a single fiber) comparable to that of steel and Kevlar [21]. As a film, BC is tough but very soft, similar to muscle tissue. Additionally, BC bears a strong resemblance to the nanofibril architecture of collagen but without immunologic reactivity, which causes a mild and benign inflammatory reaction that decreases recovery time and does not elicit a foreign body reaction [22]. Although many *in vitro* experiments have shown poor cell adhesion on the BC surface because of its superhydrophilic characteristics [23], an *in vivo* experiment in our previous research demonstrated a fast filling of the surrounding tissues into the intrapore region of the mesh after subcutaneous implantation [24]. These promising results support the idea of considering BC mesh for vaginal prostheses application.

The objective of this study was to evaluate for the first time the possibility of a BC mesh as a suitable candidate for POP treatment. In order to improve cell adhesion of BC mesh, collagen coating was performed on BC mesh. Comprehensively evaluation of the mechanical properties and *in vitro* cytocompatibility of the two kinds of meshes were performed. A one-week subcutaneous implantation in rabbit model was used to evaluate acute tissue response. Furthermore, a transvaginal ewe model was performed to evaluate the long-term (90 days) *in vivo* response of the developed meshes, based on protocol from Prof. Deprest's group [[25], [26], [27]]. We explored the implantation of BC and BCCOL vaginal meshes into 9 ewe. Chronic inflammatory host response, tissue integration and mechanical stability were accessed 90 days post-operation.

## 2 Materials and methods

### 2.1 Preparation of BC and BCCOL meshes

For the preparation of BC meshes, BC pellets (Yide Biotechnology Co., Ltd., China) were washed with a 1% NaOH solution for 24 ​h to remove bacterial residue and endotoxin and then sterilized at 121 ​°C for 15 ​min.

For the preparation of BCCOL, collagen I (extracted from bovine tendon, number molecular weight, Mn ​= ​2.0 and weight average molecular weight, Mw ​= ​3.0 ​kDa) was purchased from Shanghai Yuanye Bio-Technology Co., Ltd. (China). A 10% collagen solution was prepared in 0.1 ​mol/L acetic acid. The oven-dried BC film was immersed in collagen solution for 1 ​h at room temperature and dried at 30 ​°C for 30 ​min. The BC/collagen (BCCOL) films were cross-linked in a 5 ​wt% glutaraldehyde solution for 10 ​min at room temperature. All of the BC and BCCOL meshes were washed with ultrapure water multiple times until the electrical conductivity of the system was below 1 ​μS/cm. All BC meshes were tested for endotoxin content and proven to contain less than 0.5 EU/mL (The determination method of endotoxin level was according to United States Pharmacopeia (UPS), 2011, Chapter, Bacterial Endotoxins Test. 85>). All of the samples were dried at room temperature.

A customized pulsed CO_2_ laser cutting system (HDZ-CDC3055B, Han's Laser Technology Industry Group Co., Ltd., China, 10 ​W, λ ​= ​10,640 ​nm) was used to perforate the films to have a specified shape and pore array. For the perforated BC and BCCOL films with a thickness of 0.04 ​± ​0.01 ​mm, the laser cutting speed was 400 ​m/s, and the films were cut 10 times. A zipper-like arrangement of pores was achieved with FPC-LGS software (Han's Laser Technology Industry Group Co., Ltd., China). Based on clinical needs, the mesh was designed to have 60% porosity with pores of 2 ​mm in diameter [28,29].

### 2.2 Physicochemical characterization of the meshes

#### 2.2.1 Surface characterization

XPS (Kratos AXIS 165 electron spectrometer, Japan) was employed to acquire quantitative information on the chemical species of the surface. XPS spectra were recorded with a Kratos AXIS 165 electron spectrometer (Japan) using monochromated Al Kα X-ray irradiation generated at 350 ​W. The standard take-off angle used for analysis was 45°, which produced a maximum analysis depth in the range of 3–5 ​nm. Low-resolution survey spectra were recorded in 0.5 ​eV steps with a 187.85 ​eV analyzer pass energy from 0 to 1400 ​eV. SEM images of the samples were recorded using a scanning electron microscope (Philips XL-30, Holland). The surface topography of each sample was determined using a D3000 AFM microscope with a Nanoscope IV controller (Agilent PicoPlus). The scanning area was 1.5 ​× ​1.5 ​μm to determine roughness on the nanoscale. Root mean square (RMS) roughness values were obtained as Rq using the instrument software NanoScope Analysis.

#### 2.2.2 Mechanical properties

Tensile tests were performed on a Universal Materials testing machine (Instron, Norwood, MA, USA) equipped with a 500 ​N load cell. All the samples were soaked in phosphate-buffered saline (PBS) at room temperature for 3 days and then the surface water was absorbed using filter paper before test. Meshes that were 50 ​mm long, 10 ​mm wide and 0.04 ​mm thick were tested at a crosshead speed of 5 ​mm/min according to ASTM D5035-2011. In the case of the suture strength test, which uses the straight-across procedure [30], one 4/0 Monocryl thread was passed through the pore on most of the edge of the mesh and pulled at a continuous rate of 5 ​mm/min with a 10 ​N load cell until failure occurred. ASTM D1388-96 (R2) was used to measure stiffness using a TN11028-A Cantilever Bending Tester (TongMin, China). A strip of mesh 250 ​mm long, 25 ​mm wide and 0.04 ​mm thick was placed parallel to its long dimension, and the overhang length was used to calculate the stiffness. For clinical reference and comparison, commercially available vaginal meshes, extralight and light TiLOOP® Mesh (pfm medical titanium GmbH, Germany), which achieved good clinical effects, were used and tested as received. At least 7 samples were tested for each group.

### 2.3 *In vitro* cellular study: uterine smooth muscle cell (SMC) adhesion and the expression of collagen

The surface of the mesh is not suitable for evaluating the biocompatibility of the material since the pores are too large, causing the cells to leak during cell culture. To simplify the analysis, the materials tested in the cell experiments were integrated into membranes instead of meshes. Uterine SMCs (primary generation of rabbit uterine smooth muscle cells, RAB-iCell-f009, bs-10169R, Bioss) were cultured in Dulbecco's minimal Eagle medium containing 10% fetal bovine serum (Gibco, Thermo Fisher, Waltham, MA, USA) and 1% antibiotic-antimycotic solution (Beyotime, China) in an incubator under standard cell culture condition. The medium was changed every 2 days until 80% confluence was reached. Culture medium was used as the negative control. To evaluate cell attachment to the membranes, the membranes were placed in 96-well tissue culture plates, with seeding density of 1 ​× ​10^5^ ​cells per well. The reported results are from three different assays with triplicate samples. Cell viability was measured by Cell Counting Kit-8 (CCK-8) assay following manufacturer's instruction.

For collagen I and III staining, after 72 ​h of culture, the membranes were washed with PBS three times and fixed in 4% formaldehyde in PBS. Nuclei were visualized by staining with 4′,6-diamidino-2-phenylindole (DAPI, Beyotime, China). To visualize collagen I and III, the samples were stained with anti-collagen I and III (Gibco, USA), respectively. Diluted anti collagen I and III (dilution ratio: 1:100) directly onto each climbing tablet and put them into a wet box for incubation at 4 ​°C overnight. The diluted fluorescent secondary antibody anti Alexa fluor 647 (dilution ratio: 1:800) was added in and incubated at 37 ​°C for 30 ​min. The stained samples were evaluated by confocal laser scanning microscopy. The stained samples were evaluated by confocal laser scanning microscopy. The gene expression of collagen I and III were determined by real-time quantitative PCR. The cells were lysed using trizon lysate (CW0580S, CWBIO), and add 1 ​ml trizon per dish according to the cell volume. The RNA of the cells was extracted by using an Ultraure RNA kit (CW0581S, CWBIO). cDNA was synthesized using HiFiscript cDNA synthesis kit (CW2569S 25rxns, CWBIO). qRT-PCR was then performed using the UltraSYBR mixture Kit (CW0581S, CWBIO). Use of kits was according to the manufacturer's procedures. Primers of Collagen I (CAAGAACGGAGATGACGGA and TTTTCACCAGGGCTACCAG), Collagen III (CCTGGTTACTTCTTGCTCTGC and AATGGGATTTCTGGGTTGG), GAPDH F (GCCGCCCAGAACATCAT) and GAPDH R (TGCCTGCTTCACCACCTT) from Beyotime (China) were used for this assay. The relative changes in gene expression were analyzed with the 2 (-Delta C(T)) method. The specificity of RT-PCR was verified by analyzing melting curves and by gel electrophoresis of the amplicons.

### 2.4 *In vivo* studies

All the *in vivo* animal experiments were approved by the Institutional Animal Care and Usage Committee of the Peking University Shenzhen Institute**,** and fulfilled the guideline of the Declaration of Helsinki. All procedures were performed by a veterinary surgeon. Surgery was performed under international standard ISO 10933–6: 2007(E) (Annex B and C). Most acute inflammatory responses are always present 7 days after device implantation. Thus, rabbit model was used for evaluate the acute inflammatory. Chronic inflammatory were evaluated by ewe transvaginal implantation for 90 days.

#### 2.4.1 Subcutaneous implantation in rabbit

For short-term implantation studies, six male New Zealand white rabbits (three for each mesh group) were used in this experiment. Every two rabbits were housed in a cage with free access to food pellets and drinking water. Anesthesia was induced by inhalation of Isofluran (Baxter) through a small animal ventilator. Temgesic® (Schering-plough) (0.01 ​mg/kg) was given intramuscularly for additional pain relief. The BC-based meshes were cut into 1 ​× ​3 ​cm pieces and sterilized with epoxyethane. The skin on the abdomen of the rabbits was shaved and disinfected with iodophor. The meshes were inserted into subcutaneous pockets on the abdomen of the rabbits without fixation. All rabbits were euthanized and subjected to necropsy one week after implantation surgery. Explants were fixed in 10% neutral buffered formalin, and sections through the approximate center of the implant site were taken and embedded in paraffin.

#### 2.4.2 Long-term Ewe vaginal implantation model

For long-term implantation studies, 9 adult ewes (mean weight 55 ​± ​5 ​kg) were used in this study, one as control, 4 for each mesh group. Before the operation, the ovaries of the ewes were removed. All meshes used for the study were sterilized with epoxyethane and cut at the time of the procedure into patches of 35 ​× ​35 ​mm. Animals were anaesthetized by mask inhalation of 2.5% halothane and maintained with 1–2% halothane in a 50/20 oxygen/nitrous oxide mixture. During the implantation, each ewe was positioned in the dorsal lithotomy position (Fig. 5A). The vagina was disinfected, and retractors were used to dilate and expose the vagina. The posterior vagina was incised from the perineal body to the vaginal apex on the midline at 60 ​mm. The vaginal epithelium was then grasped on both sides, and the fibromuscular layer was sharply dissected laterally. The mesh was implanted on the midline under the tissue of the posterior wall. Meshes were smoothed to avoid any folds and then sutured with an intersuture distance of 1.0 ​cm using a continuous 4/0 Monocryl thread. Thereafter, the vaginal surface was closed with a 3/0 Monocryl running suture. After the meshes were implanted, the animals were monitored until they awoke. Catheter drainage was maintained for three days after the operation. Tilidine and cefotaxime sodium were used for analgesia and anti-infection, respectively. The Euthanasia was performed on the ewes 90 days post-operation and vaginal tissues with implanted meshes were extracted for histological analysis.

### 2.5 Histological and immunohistochemistry analysis

All specimens from rabbit and ewe models were fixed in 4% buffered formalin for 30 ​min and embedded in paraffin. Enlarged images of the histological sections were stained with HE and Masson's trichrome staining and observed using an inverted microscope with a digital camera. Whole slide images of the tissues were scanned by a PrimeHisto XE scanner (Truelab®, China). Mouse monoclonal anti-CD45 antibody (dilution 1:200, Thermo), rabbit anti-CD86 polyclonal antibody (dilution 1:200, Abcam), and mouse monoclonal anti-CD206 antibody (dilution 1:200, Novus) were used. Sirius red/fast green stain was used to differentiate between type I and type III collagen. Slides were examined by cross-polarized light microscopy with an Axioskop 40 microscope (Carl Zeiss, Thornwood, NY). When observed under cross-polarized light, the type I collagen fibers appeared bright yellow-orange, and the type III collagen fibers appeared green-blue.

### 2.6 Imaging and statistical analysis

The *in vivo* biocompatibility of the meshes was assessed according to international standard ISO 10993–6:2009 (Annex E). The scoring system was based upon the observation of high fields (400 ​× ​) and an average of five fields. Image analysis was undertaken with ImageJ (version 1.52v). Twenty photomicrographs with 400 ​× ​magnification of the implanted area of each animal were used to assessment of positive area. The results are expressed as the mean ​± ​standard error of the mean or mean ​± ​standard deviation using Origin 8.0 software. All statistical evaluation analyses were performed with SPSS 10. Significance was accepted at *p* ​< ​0.05.

## 3 Results

### 3.1 Surface properties and tensile strength of the meshes

**Fig. 1:**
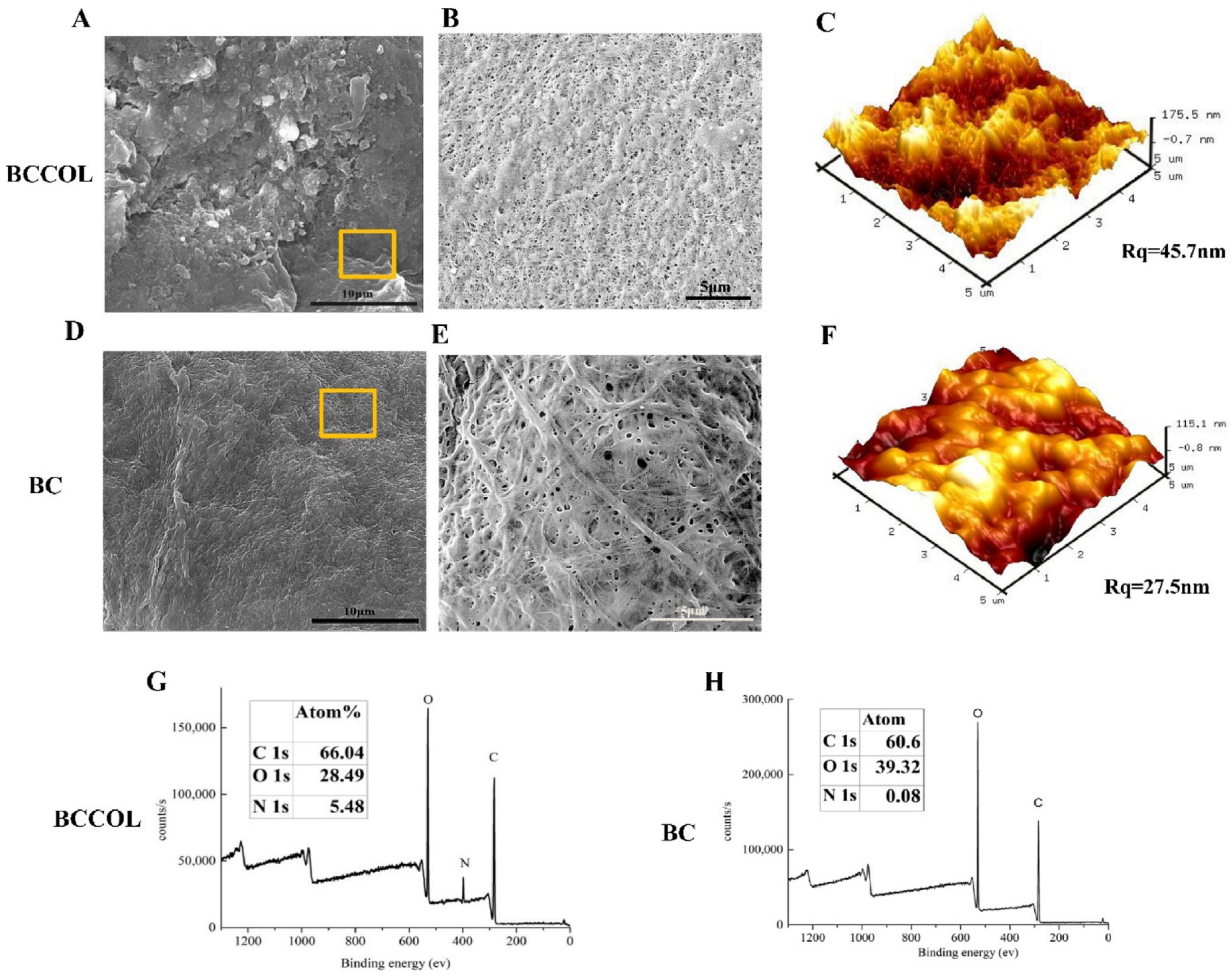
**Surface analysis of the meshes.** SEM and AFM topography images of the (**A, B, C**) BCCOL mesh and (**D, E, F**) BC mesh. Scale bar: 10 ​μm. Squares (□) indicate the magnified fields. Scale bar: 5 ​μm. XPS spectra of the (**G**) BCCOL and (**H**) BC meshes.

The surface chemical compositions and morphologies of the BC and BCCOL meshes are shown in Fig. 1. A fiber-shaped nanostructure was clearly visible on both the BC and BCCOL meshes (Fig. 1 B and **E**), which formed coarse-textured surfaces. The deposition of collagen led to a much denser and coarser structure. AFM can complement SEM with regard to morphological characterization, providing more information about nano-dimensional configurations. The surfaces of both BC and BCCOL meshes showed nodular morphologies (hills and valleys) due to the nanofibers (Fig. 1C and **F**). The nodular surface morphology of BCCOL mesh became apparent, showing some increases in roughness (Rq ​= ​45.7 ​nm) compared to that of the bare membrane (Rq ​= ​27.5 ​nm) (Fig. 1C and **F**). X-ray photoelectron spectroscopy (XPS) is the most effective technique for determining the surface chemical composition. The main spectral regions—C 1s, O 1s and N 1s—of the bare BC and coated BC meshes are shown in Fig. 1G and **H**. The C 1s peak appeared at a binding energy of 284.6 ​± ​5 ​eV^19^. The peak at 531.4 ​± ​10 ​eV corresponded to the O 1s region. The N 1s region contained a very distinct peak centered at 400.8 eV^19^, which was assigned to the cross-linked collagen on the BC mesh surface. Moreover, the XPS results revealed an increase nitrogen content (5.48%) in BCCOL mesh than in BC mesh at the surface even after repeated washings, indicating the effectiveness of the glutaraldehyde system to cross-link collagen with BC.

**Fig. 2:**
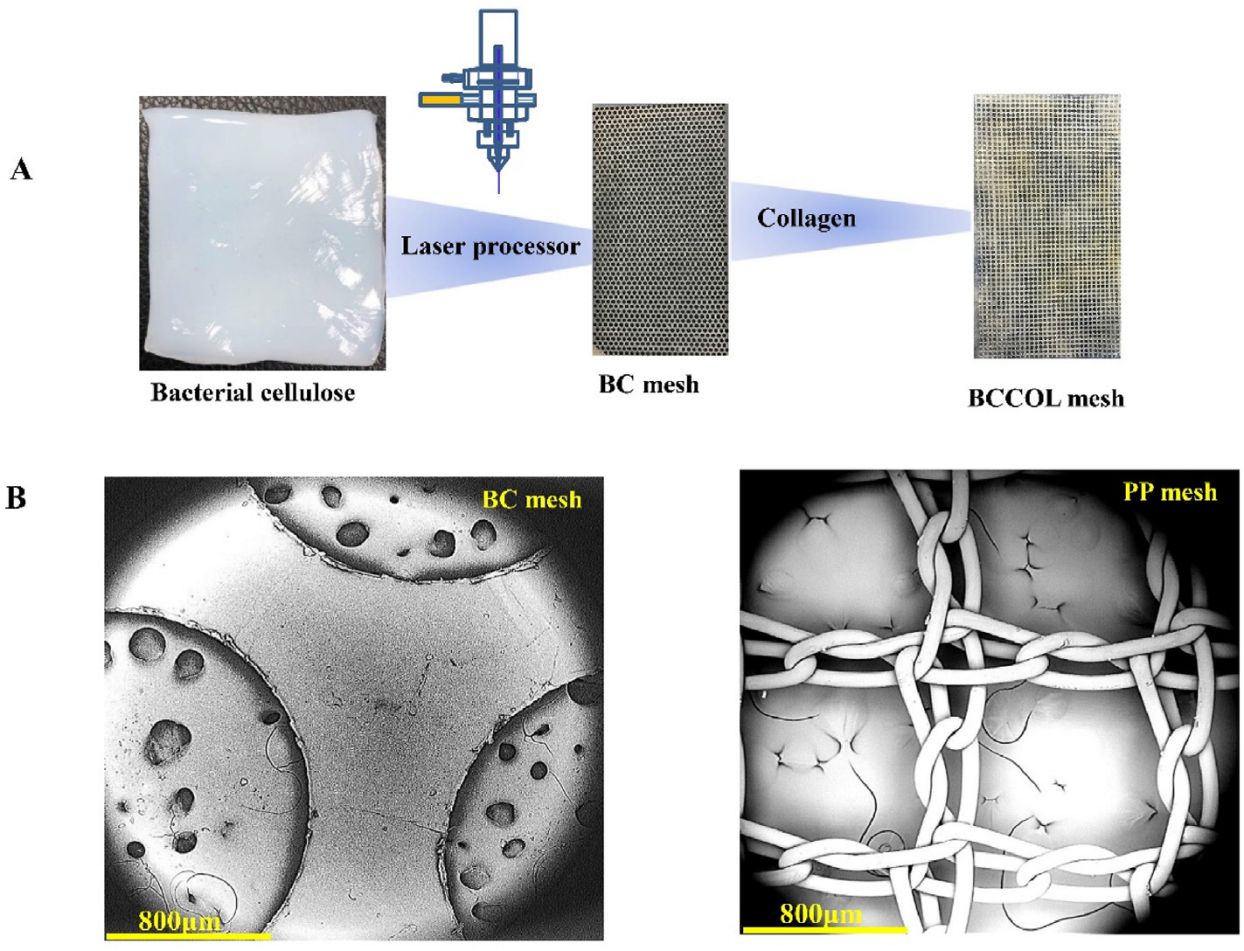
**(A)** The preparation of BC-based meshes. **(B)** The typical SEM images of BC and PP meshes. Scale bar: 800 ​μm.

Fig. 2 shows the preparation of BC-based meshes and the SEM images of BC and PP meshes. Table 1 summarizes the strength data for the four kinds of meshes. The tensile strength was reported to be 0.58 ​± ​0.07 ​MPa for vaginal tissue [31]. As expected, the BC mesh had the highest breaking strength among the meshes. The strength of the BCCOL mesh was significantly lower than that of the bare BC mesh. In the present study, the difference in suture strength was not as large as that of the breaking strength. The suture strengths of BCCOL and BC meshes were both slightly higher than that of the clinical extralight PP mesh but lower than that of the light PP mesh. Rigidity is another important parameter that impacts the handling properties of meshes. The extralight PP mesh showed the lowest flexibility. By contrast, the BC-based meshes exhibited almost three times flexibility value as the one of extralight PP.

**Table 1:** Strength characteristics of the vaginal meshes.

Sample	Breaking strength (MPa)	Suture strength (N)	Flexural rigidity (mN.cm)	Thickness (μm)
	Median (interquartile range)			
BCa)	36.5 (4.5)	6.6 (3.0)	703.0 (7.8)	40.0 (4.0)
BCCOLa)	25.5 (5.5)	7.2 (3.7)	715.0 (7.4)	40.0 (4.0)
Extralight PP mesh	10.65 (2.2)∗	6.2 (2.1)∗	202.0 (2.3)∗	28.0 (5.0)
Light PP mesh	21.0 (3.5)	7.5 (2.3)∗	679 (2.0)∗	34.0 (4.0)

∗*P* ​< ​0.05, significant difference in the mean value relative to that of the BC mesh.

a) The BC and BCCOL meshes tested in this experiment each had a pore diameter of 2 ​mm, a porosity of 60% and a water content of 70%.

### 3.2 *In vitro* cellular evaluation of the meshes

**Fig. 3:**
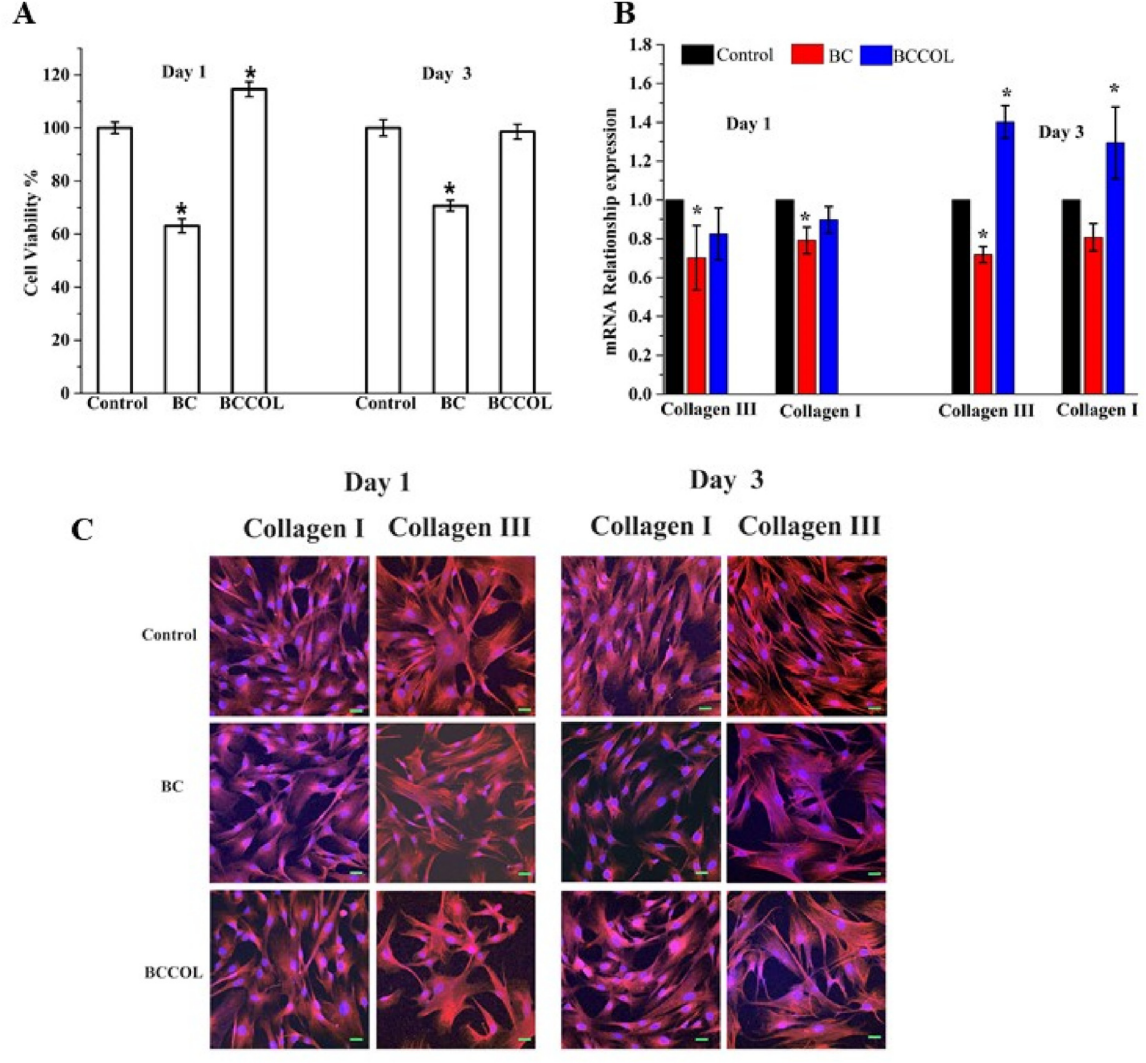
***In vitro* cellular analysis of meshes.** (**A**) Cell viability of BC and BCCOL membranes in SMCs determined by the direct contact method using CCK-8. ∗*p* ​< ​0.05 compared to BCCOL on day 3. (**B**) Real-time RT-PCR analysis of BC and BCCOL membranes regarding the mRNA expression of collagen types I and III in SMCs. ∗*p* ​< ​0.05, compared to control. (**C**) Confocal microscopy images of combined DAPI/immunofluorescence staining for collagen I and III *in vitro* after 1 and 3 days. Nuclei are visualized by staining with DAPI (blue), and collagen I and III are stained with anti-collagen I and III (red), respectively. Bar: 50 ​μm; magnification 200 ​× ​.

As anticipated, the cell viability determined by CCK-8 (Fig. 3A) was much greater on collagen-coated BC mesh than on bare BC mesh from the beginning of day 1 to day 3. However, the viability of cells on BCCOL mesh decreased from 114.58% to 98.5% after 72 ​h, while that on BC mesh increased from 63.06% to 70.6%. There were some differences in the morphologies of collagen I and III in the confocal microscopy images shown in Fig. 3C, in which collagen I appears as thinner fibers and collagen III is flakier. The cell nuclei are highlighted in blue, and clusters of cells were found on both mesh materials, BC and BCCOL meshes, after 72 ​h. The expression levels of collagen types I and III were significantly higher on the BCCOL mesh than on the BC mesh (Fig. 3B). The collagen III/I ratio on BC remained at 0.88 from day 1 to day 3, while that on BCCOL mesh slightly increased from 0.89 to 1.09.

### 3.3 Subcutaneous implantation in rabbit model

**Fig. 4:**
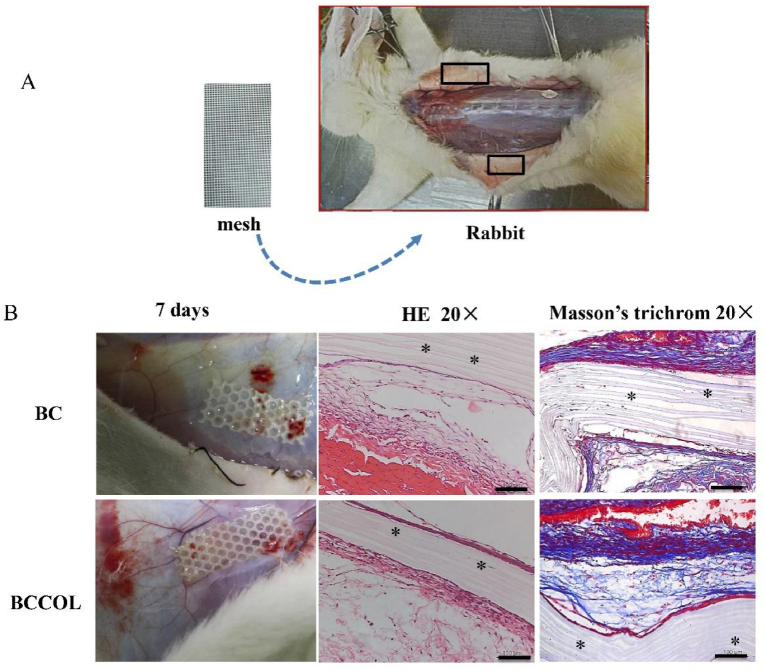
**Short-term subcutaneous implantation in rabbit mode for one week.** (**A**) Representative surgery for placing mesh in the subcutaneous pocket. (**B**) Macroscopic picture after subcutaneous implantation for one week. A representative cross section of mesh stained with H&E, and Masson's Trichrome staining. Asterisks (∗) indicates the mesh pieces; magnification 20 ​× ​.

The meshes were inserted into subcutaneous pockets on the abdomen of the rabbits (Fig. 4A). One-week post-implantation, there were no macroscopic signs of inflammation, such as redness, fever, edema or exudates, around the implanted BC-based meshes. In addition, the meshes were encapsulated by thin white connective tissue as shown in Fig. 4B. No neutrophils and other motile white cells or fragmented dead cells were observed in these experiments. The activity of the fibroblasts can be identified through Masson's trichrome staining as shown in Fig. 4B. One-week post-implantation, the histological study shows that strip-like collagen bundles (shown in blue) are deposited at the periphery of the material. The response of the tissue to the implant is summarized in Table 2, demonstrating that the BC-based meshes only showed mild host response.

**Fig. 5:**
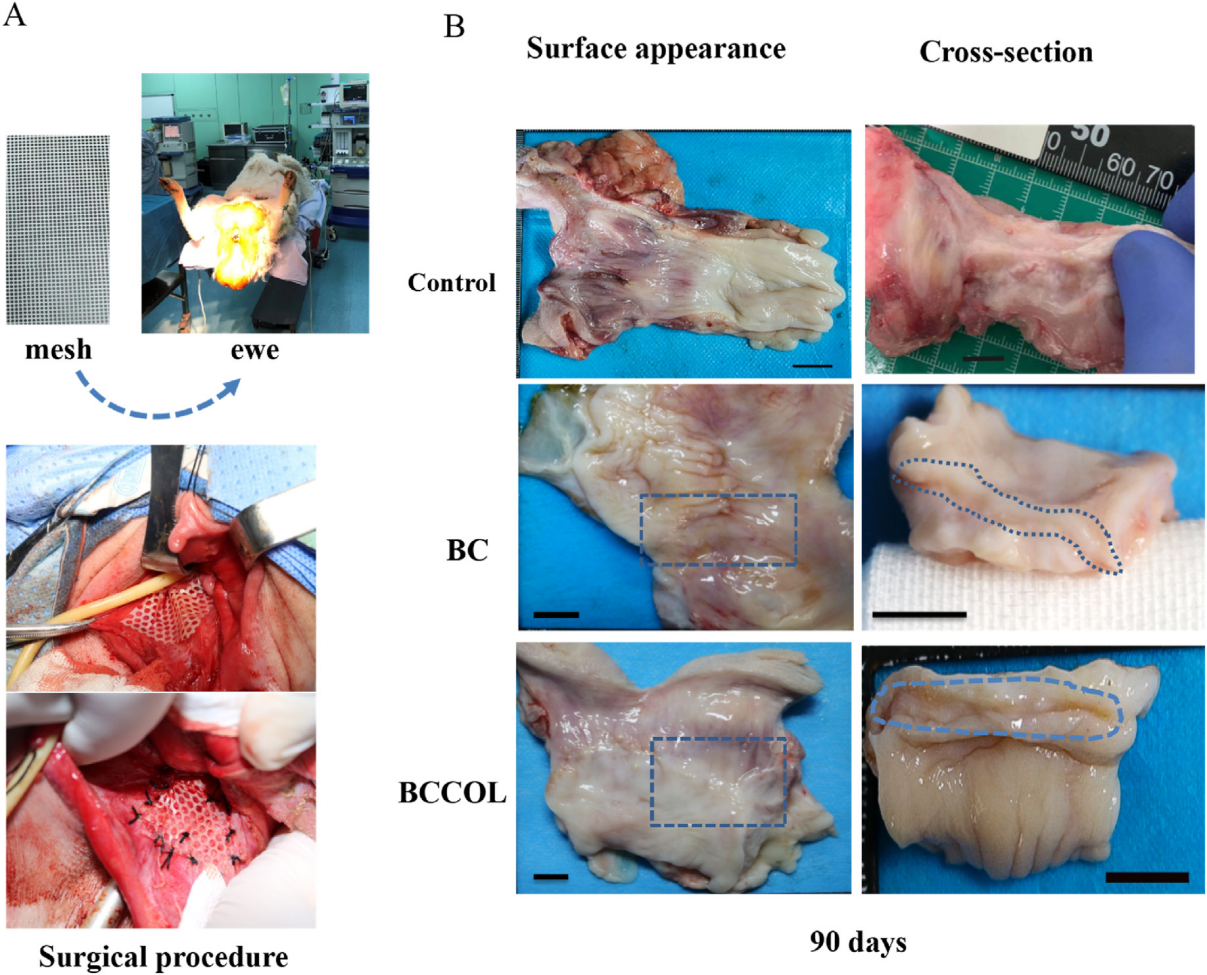
The long-term vaginal implantation in eve model. (A) Position of the ewe for vaginal access and image of the surgical procedure. (B) Gross surface and cross-section appearance of harvested control and BC-based meshes on postoperative day 90 revealing no signs of inflammation, erosion or contracture around the mesh. The intimate integration of the meshes with tissue was indicated in the blue dashed frame. Scale bar: 10 ​mm.

**Table 2:** Histological microscopic examination scores of the BC and BCCOL meshes after implantation in rabbit.a).

Sample	BC (n ​= ​3)	BCCOL (n ​= ​3)
Necrosis	0	0
Foreign body giant cells	0	0
Macrophages	0	0
Plasma cells	0	0
Lymphocytes	0.5 (0.375)	0.1 (0.75)
Polymorphonuclear cells	0	0
Neovascularization	0	0
Shrinkage of the implant	0	0
Fibrous encapsulation	1 (0.75)	2 (1.5)
Fatty infiltration	0	0

a Scoring system according to ISO 10993–6:2009 (Biological evaluation of medical devices-part 6: Tests for local effects after implantation. Annex E).

### 3.4 Long-term transvaginal implantation in Ewe model

The ewe was positioned in the dorsal lithotomy position, as shown in Fig. 5A. One mesh was implanted into the posterior wall of each ewe. Ninety days post-implantation, samples were harvested. Representative images of the macroscopic examination of the mesh and surrounding tissue are presented in Fig. 5B. Throughout the experiment, no ewes exhibited pain behavior, and none of the animals displayed visible inflammation or infection (Fig. 5B). The mesh was encapsulated and totally integrated into the host tissue, and none of the specimens exhibited erosion, exposure or contraction. Because the material and tissue are completely fused, it is hard to accurately determine whether the meshes undergo serious deformation in the body. However, by touching the specimens with the hand directly, the harvested vaginal tissues were soft and smooth without bumps. It is reasonable to speculate that there were no serious creases, deformation or folding of mesh in the body.

**Fig. 6:**
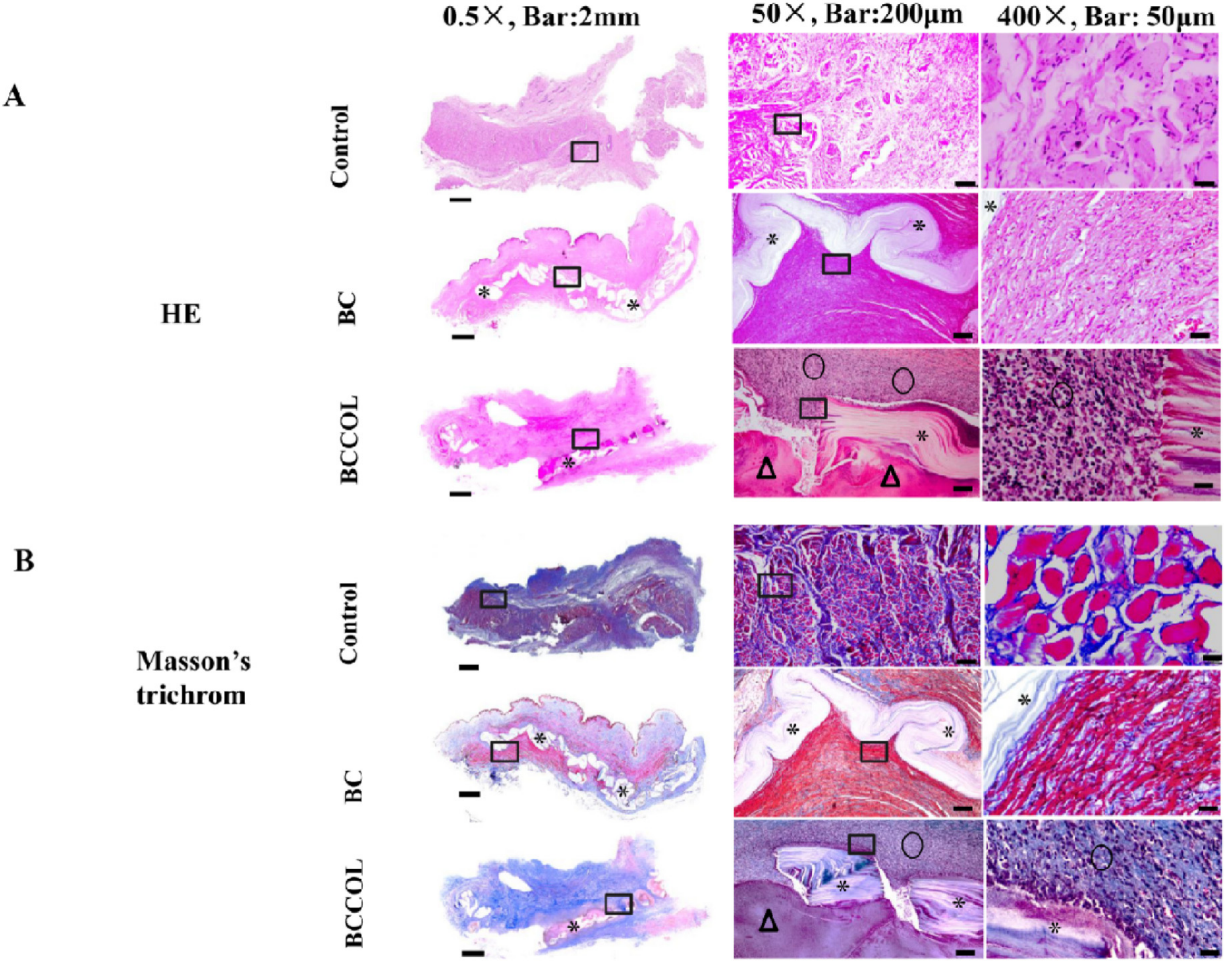
Histological analysis of the meshes at various magnifications 90 days post-implantation in ewe vaginal model. Asterisks (∗) indicate the mesh pieces. Triangles (Δ) indicate the exudate. Rings (○) indicate inflammatory cells. Squares (□) indicate the magnified fields. (A) HE images the meshes. (B) Masson's trichrome staining of the meshes. The BCCOL sample shown in this figure had the strongest inflammatory response among the four BCCOL samples. Control is vaginal tissue without mesh.

There was only 1 relatively severe inflammatory reaction from the collagen-coated BC mesh based on histological examination (Fig. 6), although there were no significant differences between the two kinds of meshes during macroscopic observation. The response of the tissue to the implant is summarized in Table 3, demonstrating that the BC mesh showed a relatively lower host response than the BCCOL mesh. The inflammatory reaction and collagen deposition were examined with hematoxylin-eosin (HE) and Masson's trichrome staining (Fig. 6). Consistent with the macroscopic results, the histological evaluation of the tissue specimens showed that the BC mesh was fully integrated into the host tissue. The deformation of the mesh is due to squeezing from tiny folds in the vaginal tissue and the active movement of the ewe. The BC mesh HE images showed that the implant was surrounded by a layer of connective tissue due to the presence of tissue cells. No inflammatory cells were found around the BC meshes (Fig. 6A). Masson's trichrome staining of the BC mesh showed that approximately two-thirds of the mesh was surrounded by red muscle fibers instead of blue collagen (Fig. 6B), demonstrating excellent biocompatibility. The same tissue response was observed in three of the BCCOL meshes. However, one BCCOL sample was less well integrated into the surrounding tissue, and more signs of exudative and inflammatory cells were observed.

**Table 3:** Histological microscopic examination scores of the BC and BCCOL meshes after implantation in ewe.a).

Sample	BC (n ​= ​4)	BCCOL (n ​= ​4)
	Median (interquartile range)	
Necrosis	0	0
Foreign body giant cells	0	0.5 (0.75)∗
Macrophages	0.5 (1.0)	0.5 (1.75)∗
Plasma cells	0	0
Lymphocytes	1 (0.75)	1 (0.5)∗
Polymorphonuclear cells	0.5 (0.35)	0.5 (0.35)
Neovascularization	0.5 (0.75)	0.5 (0.75)
Shrinkage of the implant	0	0
Fibrous encapsulation	1 (0.75)	0.5 (1.0)∗
Fatty infiltration	1 (0.75)	0.5 (0.75)

∗*p* ​< ​0.05 compared to counterpart in BC mesh.

a) Scoring system according to ISO 10993–6:2009 (Biological evaluation of medical devices-part 6: Tests for local effects after implantation. Annex E).

**Fig. 7:**
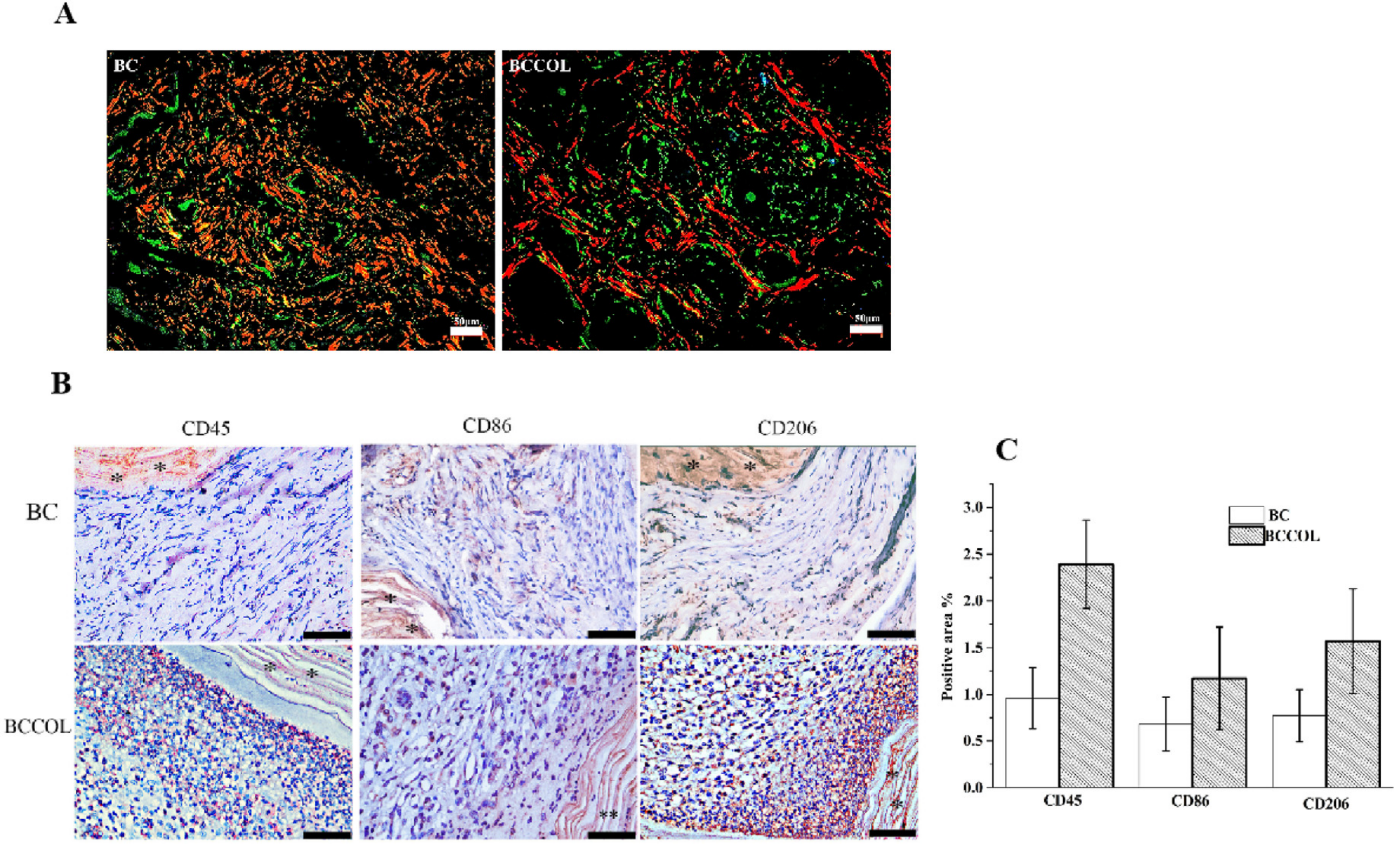
Assessment of Sirius red birefringence and immunohistochemical analysis. (A) Digital images of Sirius red/fast green-stained BC and BCCOL meshes viewed by cross-polarized microscopy. Collagen types I and III are highlighted in red and green, respectively. Bar: 50 ​μm; magnification 200 ​× ​. (B) Representative immunohistochemical staining images. Bar: 50 ​μm; magnification 400 ​× ​. (C) Bar plots showing the positive immune-stained area around the meshes. ∗*p* ​< ​0.05.

Sirius red/fast green staining can distinctly highlight collagen. Collagen types I and III, which were specifically highlighted, presented red and green colors, respectively, in the birefringent pattern, leading to easy delineation. Predictably, type I collagen was more predominant than type III collagen in the BC samples (Fig. 7A). Moreover, an increase in the number of green (type III) fibers compared with red (type I) fibers was observed in the BCCOL meshes. This trend is roughly consistent with the collagen III/I ratio results *in vitro*. However, image analyses and the quantitative assessment of collagen III/I were not performed in the present study. Because observations of the red and green color were not always clear, admixes that were shades of red and green were present. Therefore, the analytical error would be very large when assessing the collagen ratio with Sirius red/fast green staining using imaging software.

Immunohistochemistry was used to evaluate the chronic inflammatory reaction. CD45 is specifically expressed in all hematolymphoid cells and is used to detect the degree of inflammatory infiltration in tissue (Fig. 7B). No inflammatory cells were observed around the BC mesh. In the BCCOL sample, CD45-positive cells were noted, which was also evidenced by the HE staining results (Fig. 6A), with exudation. M1 macrophages expressed high levels of CD86, while M2 macrophages expressed relatively high levels of CD206. As shown in Fig. 7B, the number of CD86^−^and CD206-positive cells in the BCCOL sample was significantly higher than that in the BC samples. However, the number of CD86-positive cells was lower than that of CD206-positive cells in both samples (Fig. 7C). The results demonstrated that the BCCOL mesh displayed a higher degree of chronic inflammation than the BC mesh. However, the inflammatory cells around BCCOL meshes were predominately lymphocytes, with a lower content of macrophages, and no foreign body giant cells were present. Around the BCCOL mesh, more M2 cells were observed than M1 cells. Few macrophages were observed around the BC mesh, and those that were observed were primarily of the M2 phenotype.

## 4 Discussion

The current poor biocompatibility and host response of synthetic vaginal mesh have been recognized as the main challenges for synthetic meshes for POP application. To address this, we developed BC and collagen-coated BC meshes in this study. In comparison with clinical synthetic meshes, BC mesh exhibited extremely high breaking strength and comparable suture strength. Due to the unique nanoscale structure, the BC meshes showed excellent rigidity and conformability. In long-term ewe vaginal implantation model, there were no signs of infection, exposure or contracture. Histological evaluation showed good ingrowth of muscle tissue into the BC, without serious fibrosis. Additionally, newly formed vaginal muscle tissue surrounding the BC returned to its original physiology as muscle tissue, indicating the excellent integration of BC into the host tissues without triggering severe local inflammatory response in long-term. On the other hand, the collagen coating on BC increased cell adhesion and activity *in vitro*; however, it provided little advantage *in vivo* for long term*.* Therefore, the developed BC might be a suitable candidate mesh for POP treatment.

The reason for developing a collagen-coated BC mesh was due to the concern of poor cell adhesion onto the BC surface, which might lead to a delay in tissue integration. Cross-linking processes with 1-ethyl-3-(3-dimethylaminopropyl)carbodiimide/N-hydroxysuccinimide or genipin have been proven to be nontoxic and have been widely reported. However, the fibers cross-linked in those systems showed inadequate water stability and collapsed into films in aqueous or high-humidity environments [32]. Glutaraldehyde is a known crosslinker of hydroxyl and amine groups, which are abundant in cellulose and collagen [33,34].. In the present study, glutaraldehyde was used as a cross-linking agent, because it has been proven to be efficient and useful in previously published study [16]. Our study also confirmed its efficacy in cross-linking. The AFM, SEM and XPS results demonstrated that collagen was successfully incorporated into the BC mesh even after repeated washing and soaking. Notably, there are three kinds of cross-linking reactions in glutaraldehyde-treated systems. Namely, both the amine groups within collagen and the hydroxyl groups within the BC would react to the aldehyde groups in glutaraldehyde. Therefore, there will be bonds between BC and BC, collagen and collagen, and collagen and BC [34]. The reaction mechanism and process have been well documented [34].

In order to fully unveil the potential of the developed meshes for POP treatment, physicochemical evaluations were firstly conducted and compared with commercial meshes. Mechanical performance is a critical issue in POP mesh design. Before ingrowth of host tissue has occurred, especially in the early post-implantation period, sutures of the mesh are the primary repair component preventing failure. In the present research, both the BC and BCCOL meshes exhibited slightly higher suture strengths than the clinical PP meshes, indicating these meshes can provide sufficient strength at the suture site in the clinic*.* In addition, the BCCOL and BC meshes with 60% porosity had superior breaking strengths compared with that of the PP mesh, even in the wet state, due to their nanostructure and stiff fibrous constituents. Both the BC and BCCOL meshes showed much higher breaking strength than that of normal female vaginal pressure [35]. Moreover, our study revealed that nonstrain sutures and more stitches can effectively maintain the strength of BC-based mesh *in vivo* as no suture broke after 90 days. Although the *in vivo* mechanical change of BC-based mesh was unclear, the ingrowth of muscle tissue into the mesh may be helpful for maintaining the strength of the meshes [36].

Rigidity and conformability are important considerations in surgical mesh [[37], [38], [39]]. The rigidity of the BC-based meshes were found to be almost three times more than the one of the extralight PP mesh. The latter was found to have insufficient mesh rigidity and undergo considerable folding *in vivo* in Deprest's research [30]. Therefore, the BC meshes developed in this study possess desirable rigidity as surgical mesh. Moreover, due to its unique nanostructure and hydrogen bonds between the nanocellulose fibers and water [40], BC can absorb up to 200 times its dry mass of water [41], leading to fiber swelling and an increase in thickness. The increased thickness of the BC-based meshes favors rigidity but only has a limited influence on the softness and conformability of the mesh. In other words, the wet BC mesh can output a smooth contour that fits better to the tissue instead of self-folding or creasing, which has been proven during clinical trials in a large number of patients [42,43]. Notably, we observed in our experiments that the mesh can adhere tightly to the tissue and deform with vaginal folds during the implantation procedure (please watch the Supplementary Video about the mesh implantation procedure). The results of animal experiments also confirmed that all the harvested vaginal tissue felt smooth to the touch.

Supplementary video related to this article can be found at https://doi.org/10.1016/j.mtbio.2021.100172.

The following is the supplementary data related to this article:

Video 1 Video 1

In cellular assays, compared to the bare BC mesh, the BCCOL mesh exhibited a potentially advantageous effect on both cell viability and adhesion, showing a higher overall concentration of collagen. On an extremely hydrophilic surface, cell adhesion-mediating proteins are loosely bound, disfavouring the adhesion and spread of cells, as was observed on the bare BC membrane with relatively low cell viability and a lower overall concentration of collagen (Fig. 3). Collagen types I and III are fibrillar collagens produced mainly by SMCs, as shown in Fig. 3C. Type I collagen is composed of rigid fibrils that provide tensile strength, whereas type III collagen is immature and too weak to increase the extensibility of the tissue [44]. *In vivo* results showed that the expression of collagen III increased to a greater extent around the BCCOL samples than the BC samples, which was consistent with the *in vitro* results. Enhanced collagen III deposition is associated with inflammation [45], and new collagen deposition at the site of mesh implantation has been used as an important indicator of fibrotic changes. Therefore, it is necessary to investigate deeply the short-term and long-term host response of the BC-based meshes.

The short-term responses were conducted using a rabbit model system in which BC-based meshes were implanted subcutaneously. No inflammatory response and no foreign body reaction were observed in current study. Based on histological studies, the majority of cells around the meshes were fibroblasts, demonstrating that BC-based materials triggered very low acute inflammatory response. Just a week later, the materials were surrounded by newly formed connective tissue. It seems that both BC and BCCOL meshes are ideal for integration with the surrounding tissues.

**Fig. 8:**
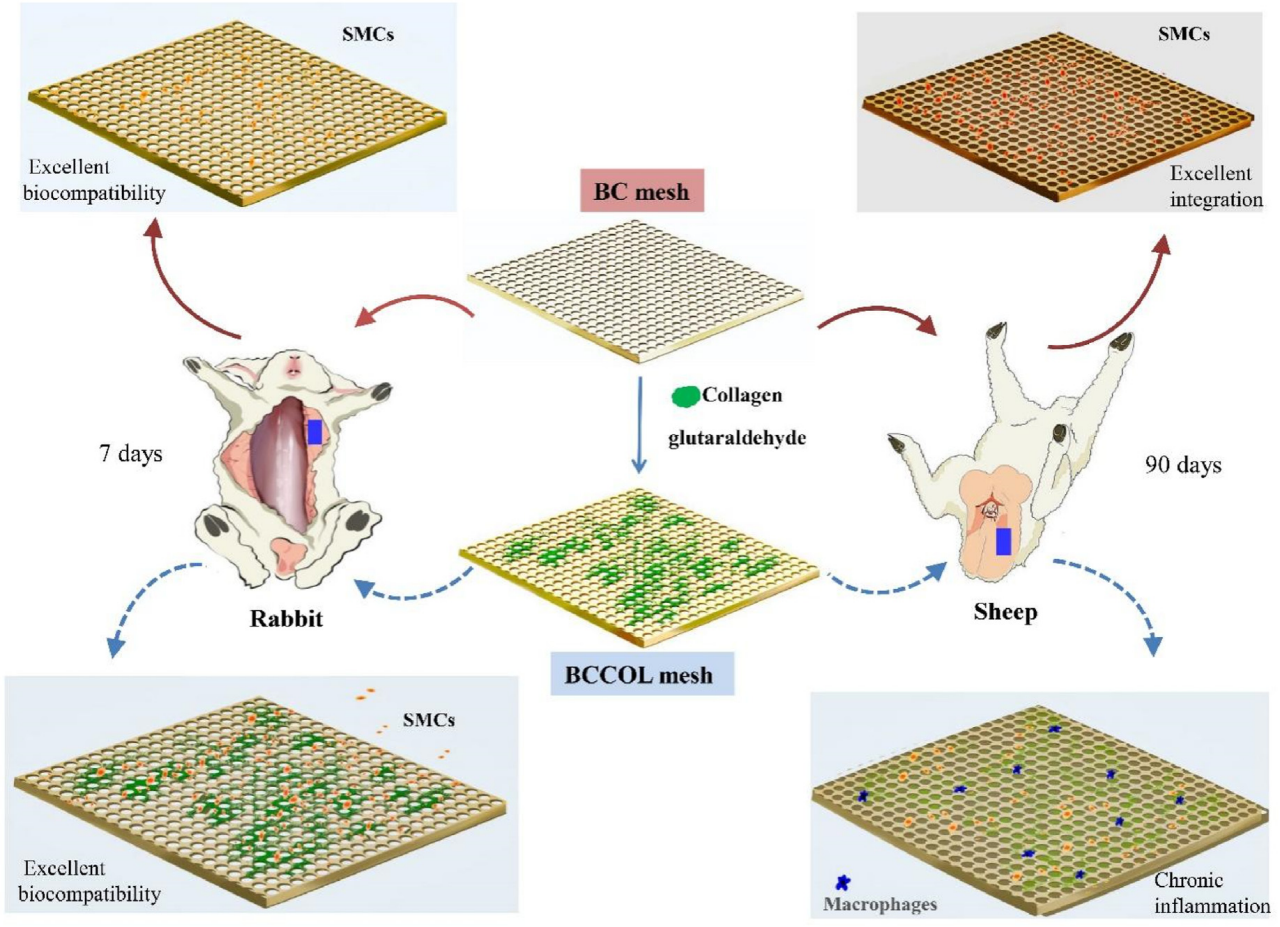
Schematic of short-term and long-term implantation studies and local tissue responses for BC-based meshes. For detail, please see the text.

For non-degradable material, long-term chronic inflammation evaluation is necessary. Inflammatory reactions are necessary for connective tissue deposition, while enhanced immunological activity may lead to postoperative complications. Chronic inflammation may create a profibrotic milieu that enables collagen production [44,46] and enhanced collagen III deposition is associated with inflammation [45]. We found that the BC mesh displayed a marked reduction in fibrosis. Only approximately 1/3 of the whole mesh was surrounded by loose and parallel collagen. While BCCOL displayed some inflammation with exudate around the mesh, the arrangement of collagen fibers presented a haphazard pattern. Most of the BC mesh clung to the irregular smooth muscle tissue (Fig. 6), demonstrating the excellent biocompatibility of the BC meshes. Collagen I showed predominance compared with collagen III (Fig. 7), inferring that the implantation site regained some tensile strength. However, no heavy fibrosis or encapsulation could be observed in either of the BC or BCCOL samples. Macrophages are the predominant cell type found at sites of chronic inflammation. There are mainly two types of macrophages, M1 and M2. M1 macrophages participate in tissue destruction and display pro-inflammatory properties, while M2 macrophages help with parasite clearance and promote tissue remodeling. Therefore, the ratio between M1 and M2 macrophages reflects tissue inflammation, healing and remodeling [42,43,45,47]. The relative quantities of lymphocytes and macrophages, which typically characterize enhanced inflammation. Compared to the BCCOL mesh, the BC mesh elicited very low cell invasion and extracellular matrix deposition. The immunohistochemistry results revealed that only a small number of macrophages were occasionally observed around the BC mesh. Furthermore, the few macrophages surrounding the meshes were predominantly of the M2 phenotype (M1/M2<1.0), which has positive effects on tissue remodeling outcomes. As polysaccharides, BC has some of the lowest reported immunological responses and excellent biocompatibility among synthetic materials [40]. BC meshes elicit very low cell invasion and extracellular matrix deposition compared to the BCCOL meshes after examination with routine stains such as HE or Masson's trichrome. The immunohistochemistry results revealed that the BC meshes displayed very low populations of macrophages. A scheme was presented to show the short-term and long-term tissue response of the BC and BCCOL meshes (Fig. 8).

Teflon mesh, which also possesses antiadhesive characteristics, is made from PTFE [6]. Because of the very low surface energy of PTFE (13.54 ​mN/m) [48], it is difficult for fibroblasts to attach and spread on its surface, disfavoring the ingrowth of fibrous tissue into the meshes. Although we were concerned about the antiadhesive properties of BC, which might fail to integrate with tissue, some good results were obtained in the present experiment. The tissue surrounding the BC mesh returned to its original physiology as muscle tissue without severe inflammatory response. There are two possible explanations for this result. First, it has been well established that cell adhesion, spreading and migration behaviors are highly dependent on protein adsorption [49]. Proteins and cells are easily adsorbed by hydrophobic surfaces due to their strong hydrophobic interaction [50]. Therefore, the cytokine (a type of protein) adhered to the hydrophilic BC surface is very weak. In order to degrade a scaffold, macrophages need to attach and adhere to the materials first [46]. This poor cell adhesion characteristic enables BC materials to present biological inertia *in vivo* without triggering a severe post-implantation inflammatory response. The second explanation is that the BC mesh possesses excellent conformability and rigidity as we discussed previously. As a super-hydrophilic material, wet BC meshes adhere tightly to the tissue and create a seamless lamination, preventing the mesh migration and favoring the deposition of collagen and wound healing.

The enhanced inflammatory response on the BCCOL mesh was probably due to the residual of the cross-linking agent glutaraldehyde. Aldehyde group released from residual glutaraldehyde during the degradation of collagen are cytotoxic and may cause calcification. In a recent study, Emmerson et al. found that poly-(amide) mesh with glutaraldehyde crosslinked gelatin coating induced poorer tissue integration, higher mesh exposure and greater inflammatory response compared to poly-(amide) mesh in an ovine vaginal implantation model [16]. This finding was in good agreement with our study. On the other hand, the current literature showed controversial finding about the role of collagen in inflammatory response. Many studies have found that collagen-coated meshes can induce a more severe inflammatory response [43,51]. Although collagen coating on synthetic materials (PP, PTFE) apparently induced a lower exposure rate or reduced chronic inflammation [[42], [52]], collagen failed to provide an advantage once coated onto BC in our experiments. It is worthy to perform in depth study to elucidate the relationship between collagen biomaterials and host response.

One critical aspect should be considered for POP mesh design is its stability *in vivo*. It did not appear that the BC mesh was undergoing degradation. Adherent macrophages and foreign body giant cells in the foreign body reaction are now known to lead to the degradation of biomaterials, with subsequent clinical device failure [47]. The active biological degradation of BC is not expected to be possible, as mammals lack the appropriate enzymes to digest plant-synthesized cellulose. Considering all of these facts, we assumed that the BC mesh can bring good mechanical support to the vaginal wall *in vivo* by encouraging collagen deposition*.*

However, our study has several limitations. There was a lack of sampling at earlier time points to assess when fibrous tissues became incorporated into most of the pores in the meshes. Another limitation was that this study did not establish the relationship between M1 macrophages and endotoxins [53]. Acetobacter is a gram-negative, α-proteobacterial genus that possesses endotoxins in its outer membrane [18,54]. Therefore, BC structures inevitably contain a large number of endotoxins. The endotoxin levels of the meshes used in the present experiment were assumed to be below the FDA mandated threshold for biological devices, which is no more than 0.5 endotoxin units (EU)/device (FDA June 2012). Endotoxins polarize macrophages towards the M1 phenotype [53]. It would be advantageous to use additional antibodies to assess M1 polarization. Actually, we found that the levels of endotoxins have very serious impacts on the biocompatibility of BC implants based on our previous study. We found that the *in vivo* biocompatibility of our previously developed BC mesh was not good (endotoxin level higher than 0.5 EU/mL, unpublished data). Therefore, we improved the process procedure to minimize the endotoxin level of the BC mesh in current study (less than 0.5 EU/mL, as mentioned in Section 2.1). Further research is needed to assess the biological effects of endotoxins by measurable M1 polarization. Last, ewe possess a thick and drooping tail that covers the introitus and anus, resulting in moisture surrounding the feces and urine. In the ewe, the urethra is located in the anterior vagina near the introitus. These moisture and urine a likely to contaminate the wound site where the mesh was implanted [55]. Postpartum ewes are also prone to endometritis and colpitis [56], which is often ignored. This disease directly affects the reliability of the experimental results. Additional experiments are needed to further develop implantation techniques and suitable animal models. Future studies should include more animals, multiple timepoints, and longer study periods to assess the potential possibility of BC for use in POP treatment without triggering a severe post-implantation local inflammatory response.

## 5 Conclusion

The present study developed a novel BC-based mesh as viable clinical alternative mesh for POP treatment. It presented superior mechanical properties to the clinical synthetic meshes, in terms of breaking strength and suture strength. It showed desirable rigidity, conformability and long-term *in vivo* biocompatibility in ewe vaginal environment, favoring *in vivo* tissue integration. Both BC and BCCOL meshes trigger very low acute inflammatory response. The BCCOL mesh displayed advantages in cell adhesion and activity *in vitro* but severe *in vivo* response during long-term implantation. Therefore, the BC mesh may be a promising candidate for clinical treatment of POP.

## Credit author statement

Chen Lai: Conceptualization, writing original draft, Funding acquisition. Shu-Jiang Zhang: Methodology, Investigation, data Formal analysis. n Xuan-Chen Chen: Investigation, Visualization. Li-Yuan Sheng: in vivo study. Tian-Wei Qi and Le-Ping Yan: Methodology, Supervision, Validation, reviewing and editing.

## Data availability

The data of this study are available from the corresponding authors on reasonable request.

## Declaration of competing interest

The authors declare that they have no known competing financial interests or personal relationships that could have appeared to influence the work reported in this paper.
